# MiR155 sensitized B-lymphoma cells to anti-PD-L1 antibody via PD-1/PD-L1-mediated lymphoma cell interaction with CD8+T cells

**DOI:** 10.1186/s12943-019-0977-3

**Published:** 2019-03-30

**Authors:** Zhong Zheng, Rui Sun, Hui-Jin Zhao, Di Fu, Hui-Juan Zhong, Xiang-Qin Weng, Bin Qu, Yan Zhao, Li Wang, Wei-Li Zhao

**Affiliations:** 1 State Key Laboratory of Medical Genomics, Shanghai Institute of Hematology, Shanghai Rui Jin Hospital, 197 Rui Jin Er Road, Shanghai, 200025 China; 2 Pôle de Recherches Sino-Français en Science du Vivant et Génomique, Laboratory of Molecular Pathology, Shanghai, China; 3 Department of Laboratory Medicine, Shanghai Rui Jin Hospital, Shanghai Jiao Tong University School of Medicine, Shanghai, China

**Keywords:** MicroRNA155, B-cell lymphoma, Tumor microenvironment, CD8+T cells, Anti-PD-L1 antibody

## Abstract

**Background:**

MicroRNAs (miRs) are involved in lymphoma progression by regulating tumor cell interaction with microenvironment. MiR155 is overexpressed in diffuse large B-cell lymphoma (DLBCL) and its biological effect on tumor microenvironment needs to be futher investigated.

**Methods:**

MiR155 was detected by quantitative real-time PCR in patients with newly diagnosed DLBCL. The mechanism of action of miR155 on lymphoma progression and tumor microenvironment was examined in vitro in B-lymphoma cell lines and in vivo in a murine xenograft model.

**Results:**

Serum miR155 was significantly elevated, correlated with tumor miR155 expression, and indicated poor disease outcome in DLBCL. MiR155 overexpression was associated with decreased peripheral blood CD8+T cells and inhibition of T-cell receptor signaling. Of note, EBV-positive patients showed higher serum miR155 than EBV-negative patients. In co-culture systems of B-lymphoma cells with immune cells, miR155 induced Fas-mediated apoptosis of CD8+T cells, which could be targeted by anti-PD-1 and anti-PD-L1 antibodies. Moreover, miR155 enhanced lymphoma cell PD-L1 expression, recruited CD8+T cells by PD-1/PD-L1 interaction and inhibited CD8+T cell function via dephosphorylating AKT and ERK. MiR155-induced AKT/ERK inactivation was more obvious in CD8+T cells co-cultured with EBV-infected B-lymphoma cells. In vivo in a murine xenograft model established with subcutaneous injection of A20 cells, PD-L1 blockade particularly retarded miR155-overexpressing tumor growth, consistent with maintenance of CD8+T cells and their function.

**Conclusions:**

As a oncogenic biomarker of B-cell lymphoma, serum miR155 was related to lymphoma progression through modulating PD-1/PD-L1-mediated interaction with CD8+T cells of tumor microenvironment, indicating the sensitivity of B-cell lymphoma to PD-L1 blockade. Also CD8+T cells could be a therapeutic mediator of immune checkpoint inhibitors in treating EBV-associated lymphoid malignancies.

**Electronic supplementary material:**

The online version of this article (10.1186/s12943-019-0977-3) contains supplementary material, which is available to authorized users.

## Background

Diffuse large B-cell lymphoma (DLBCL) represents the most common neoplastic disorder of B-lymphocytes. Although great progress has been made on DLBCL treatment, patients with relapsed or refractory disease have poor clinical outcomes, with median survival time less than 6 months [1]. Besides genetic aberrations of lymphoma cells themselves, dysfunction in immune cells of microenvironment can lead to tumor progression. However, the underlying mechanism that lymphoma cells escape from anti-tumor immune responses need to be further investigated.

Immune checkpoint inhibitors have emerged as successful therapeutic strategies for multiple aggressive cancers including lymphoma [2]. B7 homolog 1 (B7-H1), also known as programmed death ligand 1 (PD-L1), is a B7 family ligand for programmed death-1 (PD-1) and plays a key role in regulating tumor-specific T-cells [3, 4]. PD-L1 is expressed on tumor cells and controls lifespan of cytotoxic CD8+T cells when interacting with PD-1 [5, 6]. Recent studies have demonstrated that PD-L1 upregulation results in decreased activity of T-cells and subsequent tumor immune evasion [7]. Antibodies against PD-L1 enhance killing of tumor cells by protecting CD8+T cells from PD-1-mediated death [5]. Therefore, biomarkers related to tumor microenvironment may be helpful to predict clinical efficacy of anti-PD-L1 antibody on B-cell lymphoma [8, 9].

MicroRNAs (miRs) are 19- to 23-nucleotide non-coding RNA molecules and regulate gene expression by targeting mRNA at the 3′-untranslated region. In addition to their action on tumor cells mediated by oncogenes and/or tumor suppressor genes, miRs are key regulators of tumor microenvironment [10, 11]. MiR155 overexpression was previously detected by real-time PCR in tumor samples of 79 DLBCL patients and related to treatment failure [12]. However, the association of serum miR155 with clinical outcome has not yet been evaluated in a large cohort of DLBCL. In the present study, serum miR155 expression was assessed in 200 DLBCL patients and biological function of miR155 on tumor microenvironment was further revealed both in vitro and in vivo.

## Patients and methods

### Patients

Sixty patients with newly diagnosed DLBCL were treated with R-CHOP-based chemotherapy in a historical cohort of Shanghai Ruijin Hospital from 2011 to 2014, and referred to as the training cohort. The validation cohort consisted of 140 patients enrolled in a prospective, multi-center, randomized study, using R-CHOP-based chemotherapy in de novo patients (NCT01852435) treated with rituximab, cyclophosphamide, anthracycline, vincristine, and prednisone, at regular doses (doxorubicin 50 mg/m^2^, R-CHOP50, *n* = 49, or epirubicin 70 mg/m^2^, R-CEOP70, *n* = 60), or at a high dose (epirubicin 90 mg/m^2^, R-CEOP90, *n* = 31). No significant difference of clinical characteristics was observed between the training and the validation cohort (Table 1). The histological diagnosis was established according to World Health Organization (WHO) classification. One hundred healthy volunteers were referred as normal control. The study was approved by the Shanghai Rui Jin Hospital Review Board with informed consent obtained from all subjects in accordance with the Declaration of Helsinki.

**Table 1:** Clinical characteristics of the DLBCL patients and univariate analysis for predictors of PFS and OS in the training and validation cohort

Characteristics	Training cohort (*n* = 60)	Validation cohort (*n* = 140)	P value	Training cohort		Validation cohort	
				P value for PFS	P value for OS	P value for PFS	P value for OS
Age							
> 60 years	26/60 (43.3%)	49/140 (35.0%)	0.265	0.026	0.045	0.075	0.028
≤60 years	34/60 (56.7%)	91/140 (65.0%)					
Sex							
Female	24/60 (40.0%)	56/140 (40.0%)	1.000	0.261	0.541	0.396	0.918
Male	36/60 (60.0%)	84/140 (60.0%)					
Performance status (ECOG)							
0–1	51/60 (85.0%)	119/140 (85.0%)	1.000	0.100	0.048	< 0.001	0.210
2	9/60 (15.0%)	23/140 (15.0%)					
Lactic dehydrogenase							
Normal	37/60 (61.7%)	81/140 (57.9%)	0.616	0.045	0.113	< 0.001	0.011
Elevated	23/60 (38.3%)	59/140 (42.1%)					
Ann Arbor stage							
I-II	25/60 (41.7%)	72/140 (51.4%)	0.206	0.003	0.010	0.020	0.081
III-IV	35/60 (58.3%)	68/140 (48.6%)					
Extranodal involvement							
Yes	20/60 (33.3%)	33/140 (23.6%)	0.165	< 0.001	< 0.001	0.988	0.918
No	40/60 (66.7%)	107/140 (76.4%)					
Revised International Prognostic Index (R-IPI)							
Very good	10/60 (16.7%)	39/140 (27.9%)	0.230	< 0.001	0.001	0.013	0.323
Good	31/60 (51.7%)	60/140 (42.9%)					
Poor	19/60 (31.7%)	41/140 (29.3%)					
MiR155							
Low	31/60 (51.7%)	69/140 (49.3%)	0.758	0.012	0.015	0.022	0.021
High	29/60 (48.3%)	71/140 (50.7%)					

### Cells and reagents

Human B-lymphoma cell lines Farage (EBV+), DB (EBV-), EBV-producing marmoset B-cell line B95–8, and murine B-lymphoma cell line A20 were obtained from American Type Culture Collection (Manassas, VA, USA). Peripheral blood mononuclear cells (PBMCs) were isolated from peripheral blood with ficoll using density gradient centrifugation. Then ficoll medium interface was carefully removed, washed with salt-buffered solution, then centrifuged, leaving purified PBMCs. To freeze, freshly isolated PBMCs were resuspended to 5 × 10^6^ cells/mL in freezing medium containing 10% DMSO and 40% fetal bovine serum (FBS) in RPMI-1640 medium, and placed inside a freezing container at − 80 °C overnight. The following day, samples were moved to a liquid nitrogen tank for long-term storage. Cells were cultured in humidified atmosphere of 95% air and 5% CO_2_ at 37 °C. Anti-human PD-L1 antibody and anti-human PD-1 antibody were from Innovent (Suzhou, China). Anti-mouse PD-L1 antibody Invivomab was from Bio X Cell (West Lebanon, NH, USA).

### Serum and tissue miR155 assessment

Two hundred patients with newly diagnosed DLBCL and one hundred healthy controls were included in the study. Total serum miRNA was extracted using miRNeasy Serum/Plasma Kit (Qiagen, Valencia, CA, USA). MiR155 was measured by real-time quantitative RT-PCR using miScript reverse transcription Kit, using hsa-miR155 primer (MS00031486, Qiagen) and miScript SYBR Green PCR Kit (Qiagen). MiR39 (MS00019789, Qiagen) was used as endogenous control and DB cells for calibration. Total tissue miRNA was extracted using Trizol agent (Invitrogen, Carlsbad, CA, USA). RNU6 (MS00033740, Qiagen) was used as endogenous control and DB cells for calibration. The reactions were analyzed on 7500HT Fast Real-time PCR system (Applied Biosystem, Carlsbad, CA, USA). Real-time PCR was performed under the following conditions: 95 °C 15 min; 94 °C 15 s, 55 °C 30 s, and 70 °C 30 s (40 cycles). A relative quantification was calculated using the ^2-ΔΔ^CT method.

### Enzyme-linked immunosorbent assay

Serum IFN-γ was quantified by enzyme-linked immunosorbent assay using Human IFN-γ Cytokine Kit (Origene, Rockville, MD, USA) according to the manufacturer’s instructions.

### EBV DNA quantification

DNA was extracted from cryopreserved pre-treatment serum using the QIAamp DNA Mini Kit (Qiagen, Valencia, CA, USA) according to the manufacturer’s instructions. Quantification of EBV-specific sequences was performed by real-time quantitative PCR with 7500HT Fast Real-time PCR system (Applied Biosystem) using EBV PCR Fluorescence Quantitative Diagnostic Kit (DaAn Gene Co, Sun Yat-sen University, China). The copy number of EBV DNA in each sample was calculated from a standard curve with a cut-off value of 5 × 10^3^ copies/ml in serum.

### In vitro co-culture system

Transwell cell culture chambers (8 μM, Millipore Corporation, Billerica, MA, USA) were used for co-culture assay. In the co-culture system, lymphoma cells were plated on the upper chamber, with immune cells on the lower chamber, allowing direct contact of lymphoma cells with immune cells. Immune cells were mononuclear cells isolated from peripheral blood of healthy volunteers using Ficoll by density gradient centrifugation.

### Flow cytometry

Farage and DB cells were sorted by EasySep™ Human CD20+ Cell Isolation Kit, CD8+T cells by EasySep™ Human CD8+T Cell Isolation Kit (STEMCELL, Vancouver, BC, Canada). Purity of the sorted populations was greater than 98%. Fas expression on CD8+T cells was assessed using anti-Fas antibody as the primary antibody and goat anti-mouse IgG H&L (Abcam) as the secondary antibody. The median fluorescent intensity (MFI) was measured by flow cytometry. Cell apoptosis was assessed using Annexin V-FITC Apoptosis Kit (Becton Dickinson, Franklin Lakes, NJ, USA) according to the manufacturer’s instructions.

### Cell transfection

DB cells were transfected with miR155 mimics (Riobio, Guangzhou, China) or negative control (Riobio) using lipofectamine 2000 (Invitrogen) following the manufacturer’s instruction. For knockdown assay, Farage cells were transfected with miR155 inhibitor or control inhibitor (Riobio) using lipofectamine 2000.

### Lentivirus packaging and transfection

To overexpress miR155 in A20 cells, purified plasmids GV369-miR155 or control vector GV369-CON were transfected into HEK-293T cells with package vectors using lipofectamine 2000. The supernatant of HEK-293T cell culture was then condensed to a viral concentration of approximately 3 × 10^8^ transducing units/ml. The lentiviral particles were incubated with A20 cells for 8 h. The stably transfected cells were selected by green fluorescence protein.

### Luciferase report assay

Total cDNA from HEK-293T cells was used to amplify the 3’UTR (1335–1441 bp) of PD-L1, forward primer: 5′-ATCTGGTTCCGCGTGGATGAAGGGAGACAGCAGACATCTGAATG-3′; reverse primer: 5′-TCACGATGCGGCCGCTCGAGTATTCACAGGCAAAGTAGTCCTTCAAG-3′. 3′-UTR (2587-2593 bp) of PD-L1, forward primer: 5′-ATCTGGTTCCGCGTGGATCCATGAAGGGAGACAGCAGACATCTGAATG-3′; reverse primer: 5′-TCACGATGCGGCCGTCGAGCTATTCACAGGCAAAGTAGTCCTTCAAG-3′. The BamHI and XhoI restriction enzyme sites were used. HEK-293T cells were seeded in 24-well plates and co-transfected with 100 nM of miR155 mimics, 100 ng/ml UTR (1335-1441 bp) or UTR (2587-2593 bp) luciferase reporter construct and 10 ng/ml luciferase reporter using lipofectamine 2000. Cells were collected 24 h after transfection, using the Passive Lysis Buffer (30 μl per well) provided as part of the Dual-Luciferase Reporter Assay System Kit (Promega, Madison, WI, USA). Firefly and Renilla luciferase activities were examined by the Dual-Luciferase Reporter Assay System and detected by a Centro XS3 LB960 Luminometer (Berthold).

### Western blot

CD8+T cells were sorted by EasySep™ Human CD8+T Cell Isolation Kit (STEMCELL) for western blot. Cells were collected and lysed in 200 μL lysis buffer (Sigma Aldrich, Shanghai, China). Protein lysates (20 μg) were electrophoresed on 10% sodium dodecylsulfate polyacrylamide gels and transferred to nitrocellulose membranes. Membranes were blocked with 5% non-fat dried milk and incubated overnight at 4 °C with appropriate primary antibody, followed by horseradish peroxidase-linked secondary antibody. The immunocomplexes were visualized using chemiluminescence phototope-horseradish peroxidase Kit. Antibodies against phosphorylated-AKT (p-AKT, 4060), AKT (2920), phosphorylated-ERK (p-ERK, 9101), and ERK (4695) were from Cell Signaling Technology (Danvers, MA, USA). β-actin (HRP-6008) was from Proteintech (Manchester, UK) to ensure equivalent loading of cell protein.

### Immunofluorescence assay

Immunofluorescence assay was performed on methanol-fixed cells or 5 μm-frozen sections using antibody against PD-L1 (1:100, Cell Signaling), PD-1 (1:100, Cell Signaling), p-AKT (1:100, Cell Signaling) and p-ERK (1:100, Cell Signaling). Texas red conjugated donkey anti-rabbit IgG antibody (ab150075) and FITC-conjugated goat anti-mouse IgG (ab6785) were used as the secondary antibody.

### RNA sequencing and bioinformatics analysis

Among 72 patients, RNA was extracted using PAXgene Blood miRNA Kit from blood samples. Globin RNA was removed using Globin-Zero Gold rRNA Removal Kit for RNA from blood samples. Following extraction, RNA quantity was evaluated on Nanodrop and the integrity of total RNA using RNA 6000 Nano Kit on Aligent 2100 Bioanalyzer. RNA library was constructed using TruSeq RNA Sample Preparation Kit. The poly-A containing mRNA molecules were purified using oligo-dT attached magnetic beads. Following purification, the mRNA was fragmented into small pieces using divalent cations under elevated temperature. The cleaved RNA fragments were copied into first strand cDNA using reverse transcriptase and random primers, followed by second strand cDNA synthesis using DNA Polymerase I and RNase H. The cDNA fragments went through an end repair process, the addition of a single ‘A’ base, and ligation of the adapters. The products were purified and enriched with PCR to create the final cDNA library. The clusters of the cDNA library were generated on the flow cell using TruSeq PE Cluster Kit and HiSeq PE flow cell and sequenced on HiSeq 2000 system using TruSeq SBS Kit.

The mean reads counts of each sample was 91,630,631 (range 83,008,592~117,000,000), with an average of 90.9% Q30 Bases (range 87.3%~ 93.0%). Reading pairs were aligned to Refseq hg19 (downloaded from UCSC Genome Browser, http://hgdownload.soe.ucsc.edu/) by STAR (v2.5.2b) according to the Genome Analysis Toolkit (GATK, v3.7.0) recommended pipeline. Transcript counts table files were generated by the HTSeq using the GENCODE annotation database and processed with the BAM files generated by Hisat2. Limma version 3.34.9 were used to normalize the raw reads and obtain differentially expressed genes (DEGs). DEGs were then analyzed by the Database for Annotation, Visualization and Integrated Discovery (DAVID) v6.8 (https://david.ncifcrf.gov/) and were enriched in Kyoto Encyclopedia of Genes and Genomes (KEGG) pathways. Heatmap was generate by pheatmap version 1.0.10. Gene Set Enrichment Analysis (GSEA) was performed using the GSEA (v2.2.3, http://software.broadinstitute.org/gsea/downloads.jsp) with MSigDB-curated gene sets (c2.cp.kegg.v6.2.symbols.gmt).

### Establishment of EBV-infected cells

EBV virion was prepared from an EBV-producing cell line B95–8. Culture supernatant of B95–8 was divided into 1 ml aliquots in a 1.5 ml tube, and centrifuged at 14000 rpm for 90 min at 4 °C. After centrifuge, 900 μl of supernatant was removed, and the remained 100 μl of aliquot at the bottom was mixed by pipetting and then collected. The collected aliquot was diluted to make a final volume of 5 ml. The EBV-containing supernatant was stored at − 80 °C with 1 ml in each tube. DB cells (1 × 10^6^) were exposed to 1 ml of the supernatant for 72 h at 37 °C. Then, the cells were washed once with PBS and cultured every 2 or 3 days.

### Murine model

To test the in vivo efficiency of anti-PD-L1 antibody, BALB/c mice (4 week-old, obtained from Shanghai Laboratory Animal Center, Shanghai, China) were injected with 1 × 10^7^ A20 cells into the right flank. Treatments started after tumor became about 0.5 cm × 0.5 cm in surface (Day 0). Anti-mouse PD-L1 antibody Invivomab were injected at a dose of 200 μg per mouse, three times each week for two weeks. Tumor volumes were calculated as 0.5 × a (length) × b (width) ^2^.

### Statistical analysis

Difference of miR155 expression among groups was calculated using Mann-Whitney U test. Progression-free survival (PFS) was calculated from the date when treatment began to the date when the disease progression was recognized or the date of the last follow-up. Overall survival (OS) was calculated from the date of diagnosis to the last follow-up or the date of death. Univariate hazard estimates were generated with unadjusted Cox proportional hazards models. Covariates demonstrating statistical significance with *P* values < 0.05 on univariate analysis were included in the multivariate model. In vitro experimental results were expressed as mean ± S.D. of data obtained from three separate experiments and determined by t-test to compare variance. All statistical procedures were performed with the SPSS version 20.0 statistical software package or GraphPad Prism 5 software. *P* < 0.05 was considered statistically significant.

## Results

### Serum miR155 was significantly elevated in DLBCL and indicated lymphoma progression

**Fig. 1:**
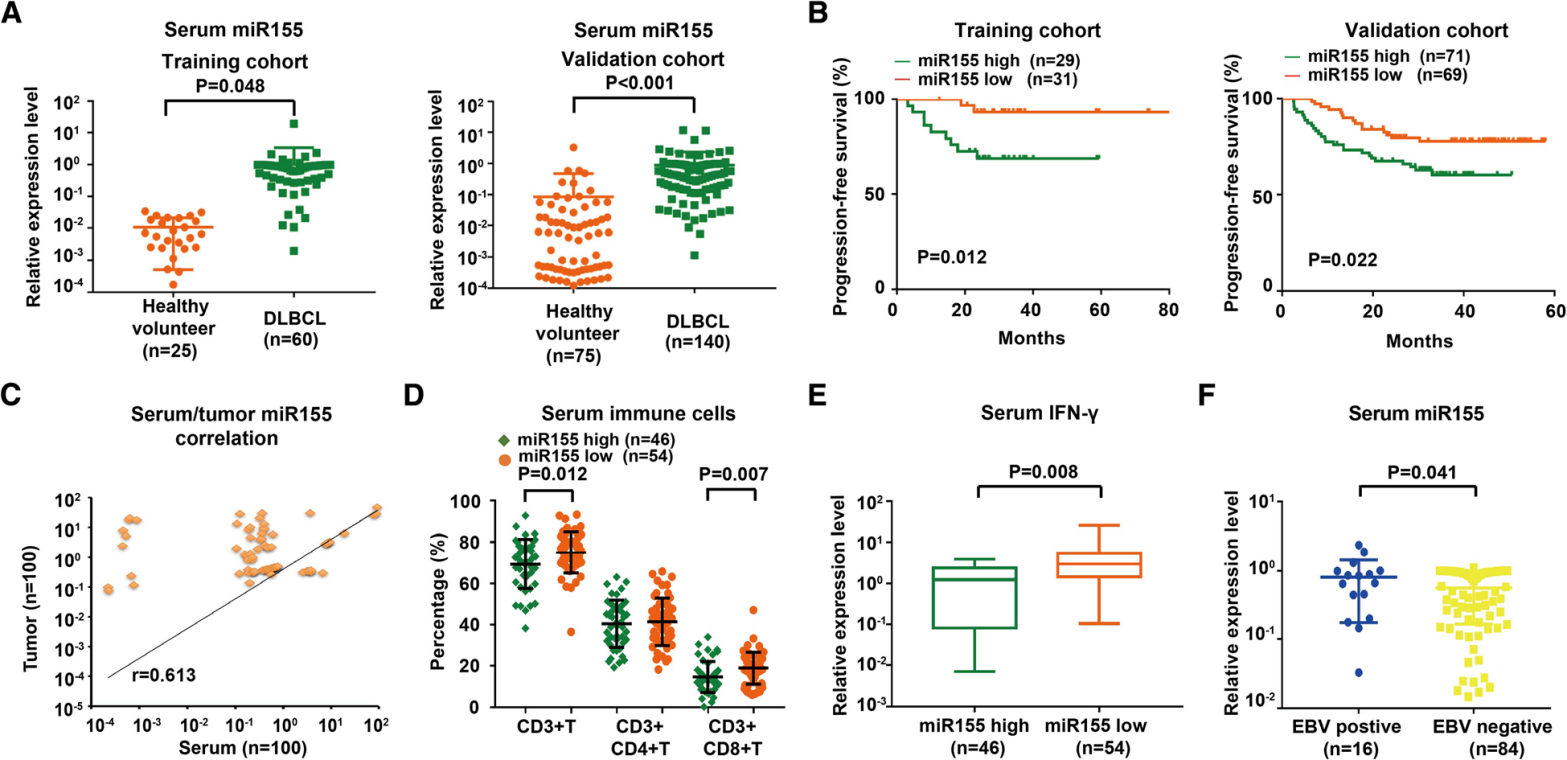
Serum miR155 was significantly elevated in DLBCL and indicated lymphoma progression. **a** As detected by real-time quantitative PCR, serum miR155 was higher in DLBCL patients than in health volunteers both in the training cohort and validation cohort. The relative expression level of each patient was calculated based on the lowest expression value. **b** Patients with high miR155 expression had significantly shorter progression-free survival time than those with low miR155 expression both in the training cohort and validation cohort calculated by survival analysis using SPSS version 20.0 statistical software. **c** A significant correlation was observed between serum and tumor miR155 expression level. Correlation coefficient was determined by Pearson correlation coefficient analysis via GraphPad Prism 5 software. **d** Patients with high miR155 expression displayed decreased serum CD3+T cells and CD3+CD8+T cells, as compared to those with low miR155 expression by analysing the peripheral blood immune cells using flow cytometry. **e** As revealed by enzyme-linked immunosorbent assay, serum IFN-γ level was higher in low miR155 group than in high miR155 group. **f** Serum miR155 was higher in EBV-positive patients than EBV-negative patients. EBV positivity was confirmed through EBV DNA quantification assay. The relative expression level of each patient was calculated based on the lowest expression value

Clinical characteristics of the DLBCL patients and univariate analysis for predictors of PFS and OS in the training and validation cohort were listed in Table 1. Comparing with healthy volunteers, serum miR155 was increased in DLBCL patients both in the training and validation cohort (*P* = 0.048 and *P* < 0.001, respectively, Fig. 1A). The median expression of miR155 was 0.660 in DLBCL. The patients with miR155 expression level over and equal to the median value were regarded as high miR155 group, while those below to the median value were included into low miR155 group. In the training cohort, the median follow-up time was 25.3 months (range, 6.1–80.8 months). The 2-year PFS and OS of the patients were 81.3 and 88.0%, respectively. By univariate analysis (Table 1), the 2-year PFS were 68.6% for patients with high miR155 expression and 93.2% for patients with low miR155 expression (*P* = 0.012, Fig. 1B left panel). By multivariate analysis, when the R-IPI was controlled, the presence of miR155 expression was an independent prognostic factor for PFS (*P* = 0.013) (Table 2). In the validation cohort, the median follow-up time was 35.0 months (range, 2.7–58.0 months). By univariate analysis (Table 1), the 2-year PFS and OS of the patients were 74.1 and 87.7%, respectively. The 2-year PFS was 67.4% for patients with high miR155 expression and 81.1% patients with low miR155 expression (*P* = 0.022, Fig. 1B right panel). MiR155 expression was associated with shorter PFS controlled by R-IPI in multivariate analysis (*P* = 0.013) (Table 2).

**Table 2:** Multivariate analysis of predictors of progression-free survival in patients with DLBCL controlled by Revised International Prognostic Index

Variable	HR	Traning cohort (95% CI)	P value	HR	Validation cohort (95% CI)	P value
R-IPI	12.087	2.684–54.472	0.001	1.916	1.261–2.911	0.002
miR155	7.035	1.497–33.053	0.013	2.230	1.183–4.202	0.013

A significant correlation between serum and tumor miR155 expression was observed by Pearson correlation coefficient analysis (r = 0.613, Fig. 1C). Moreover, high miR155 group displayed lower peripheral blood CD3+T cells and CD3+CD8+T cells than low miR155 group (*P* = 0.012 and *P* = 0.007, Fig. 1D). IFN-γ was one of the main cytokines secreted by CD8+T cells in tumor microenvironment. Serum IFN-γ level was significantly reduced in high miR155 group, as compared to low miR155 group (*P* = 0.008, Fig. 1E). Of note, miR155 levels were significantly higher in the EBV-positive patients than in the EBV-negative patients (*P* = 0.041, Fig. 1F). Accordingly, EBV+ B-lymphoma cell line Farage had high miR155 expression, while EBV- B-lymphoma cell line DB had low miR155 expression.

**Fig. 2:**
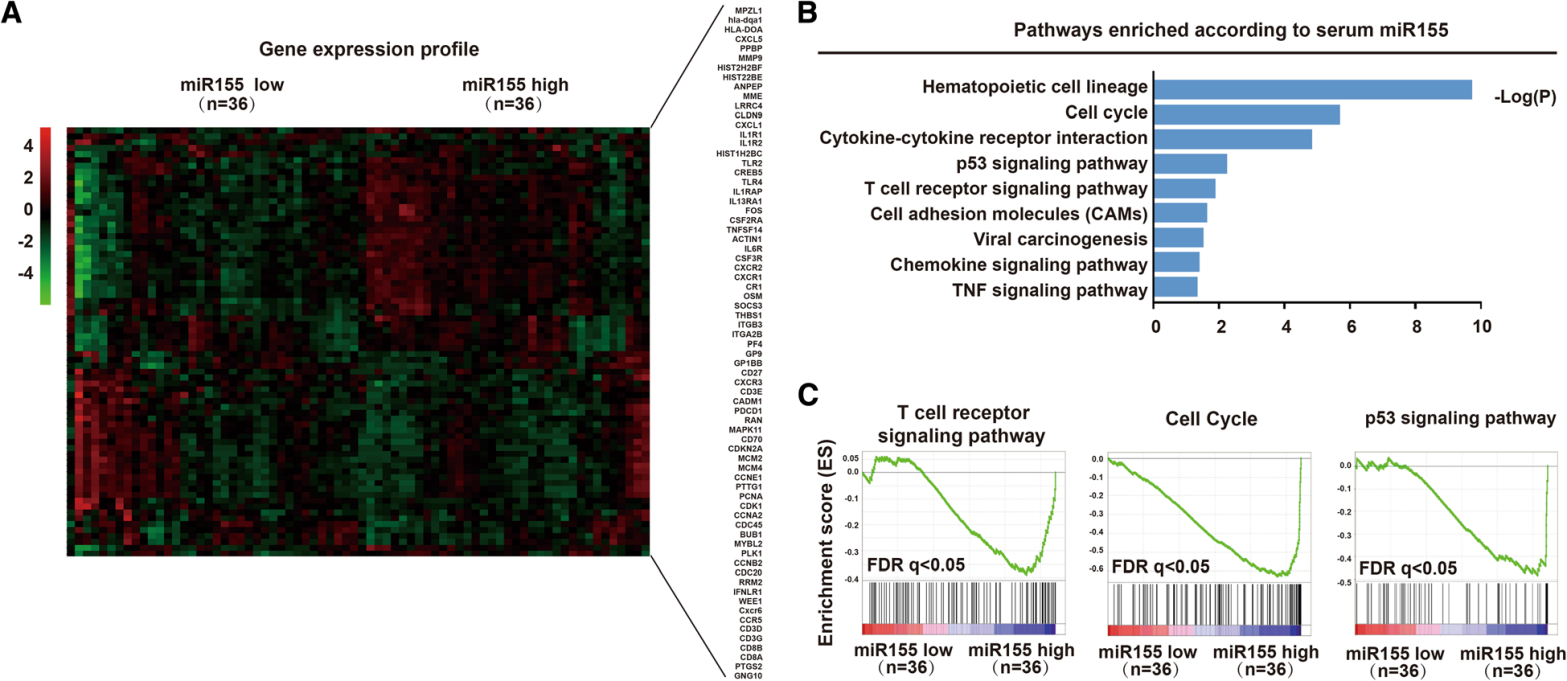
MiR155 affected cell signaling pathways in DLBCL. **a** Heat map revealed differentially expressed gene between high miR155 group and low miR155 group. **b** Dysregulated cell signaling pathways were enriched by KEGG pathway analysis. **c** T-cell receptor signaling pathway, cell cycle pathway, and P53 signaling pathway were revealed by GESA analysis and significantly altered in high miR155 group than low miR155 group

As plotted by heatmap (Fig. 2A), RNA sequencing was performed on blood samples of 72 DLBCL patients and 73 lymphoma-related genes were differentially expressed between high and low miR155 group. Multiple signaling pathways enriched by Kyoto Encyclopedia of Genes and Genomes included hematopoietic cell lineage, cell cycle pathway, cytokine-cytokine receptor interaction, p53 signaling pathway, T-cell receptor signaling pathway, cell adhesion molecules pathway, viral carcinogenesis pathway, chemokine signaling pathway and TNF signaling pathway (Fig. 2B). Gene set enrichment analysis revealed that miR155 was closely related to T-cell receptor signaling pathway, cell cycle pathway and p53 signaling pathway (Fig. 2C).

These data confirmed that miR155 contributed to lymphoma progression through altering CD8+T cells in DLBCL.

### MiR155 induced interaction of B-lymphoma cells with CD8+T cells and Fas-mediated CD8+T cell apoptosis

**Fig. 3:**
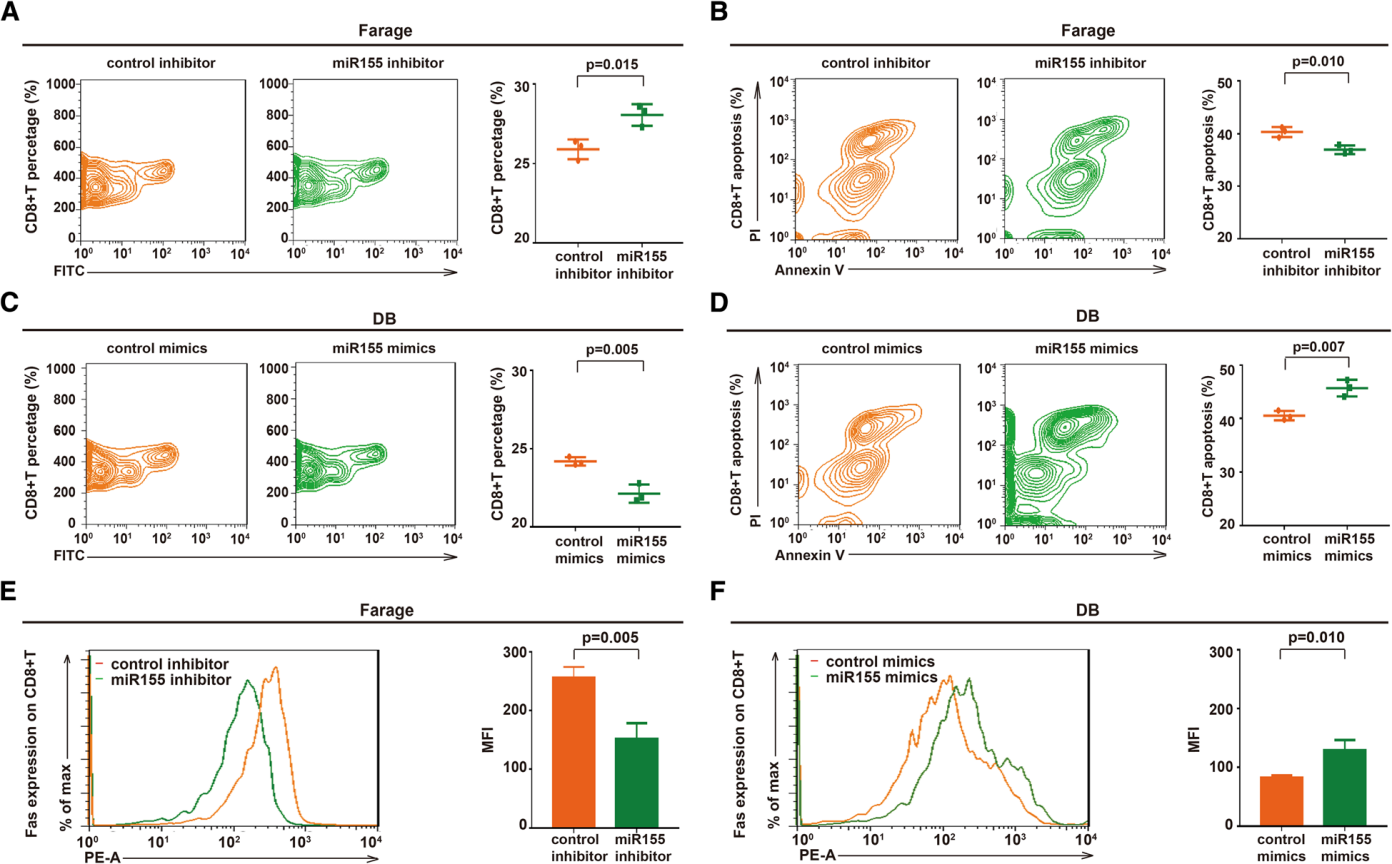
MiR155 induced interaction of B-lymphoma cells with CD8+T cells and Fas-mediated CD8+T cell apoptosis. **a** and **b:** Percentage of CD8+T cells (**a**) was increased and CD8+T cell apoptosis (**b**) was inhibited when co-cultured with Farage cells transfected with miR155 inhibitor. **c** and **d:** Percentage of CD8+T cells (**c**) was decreased and CD8+T cell apoptosis (**d**) was enhanced when co-cultured with DB cells transfected with miR155 mimics. **e** and **f**: Fas expression of CD8+T cells was downregulated when co-cultured with Farage cells transfected with miR155 inhibitor (**e**), which was upregulated when co-cultured with DB cells transfected with miR155 mimics (**f**)

To determine the biological function of miR155 on tumor microenvironment of DLBCL, EBV+ Farage cells were transfected with miR155 inhibitor and EBV- DB cells with miR155 mimics. To mimic the in vivo situation, lymphoma cells were co-cultured with immune cells and CD8+T cells were sorted from the co-culture systems. Comparing with control cells, knockdown of miR155 in Farage cells significantly increased the percentage of CD8+T cells (*P* = 0.015, Fig. 3A) and inhibited CD8+T cell apoptosis (*P* = 0.010, Fig. 3B). In contrary, ectopic expression of miR155 in DB cells decreased the percentage of CD8+T cells (*P* = 0.005, Fig. 3C) and enhanced CD8+T cell apoptosis (*P* = 0.007, Fig. 3D). In consistent with CD8+T cell apoptosis, Fas expression on CD8 + T cells was decreased when co-cultured with Farage cells transfected with miR155 inhibitor (P = 0.005, Fig. 3E), while increased when co-cultured with DB cells transfected with miR155 mimics (P = 0.010, Fig. 3F).

**Fig. 4:**
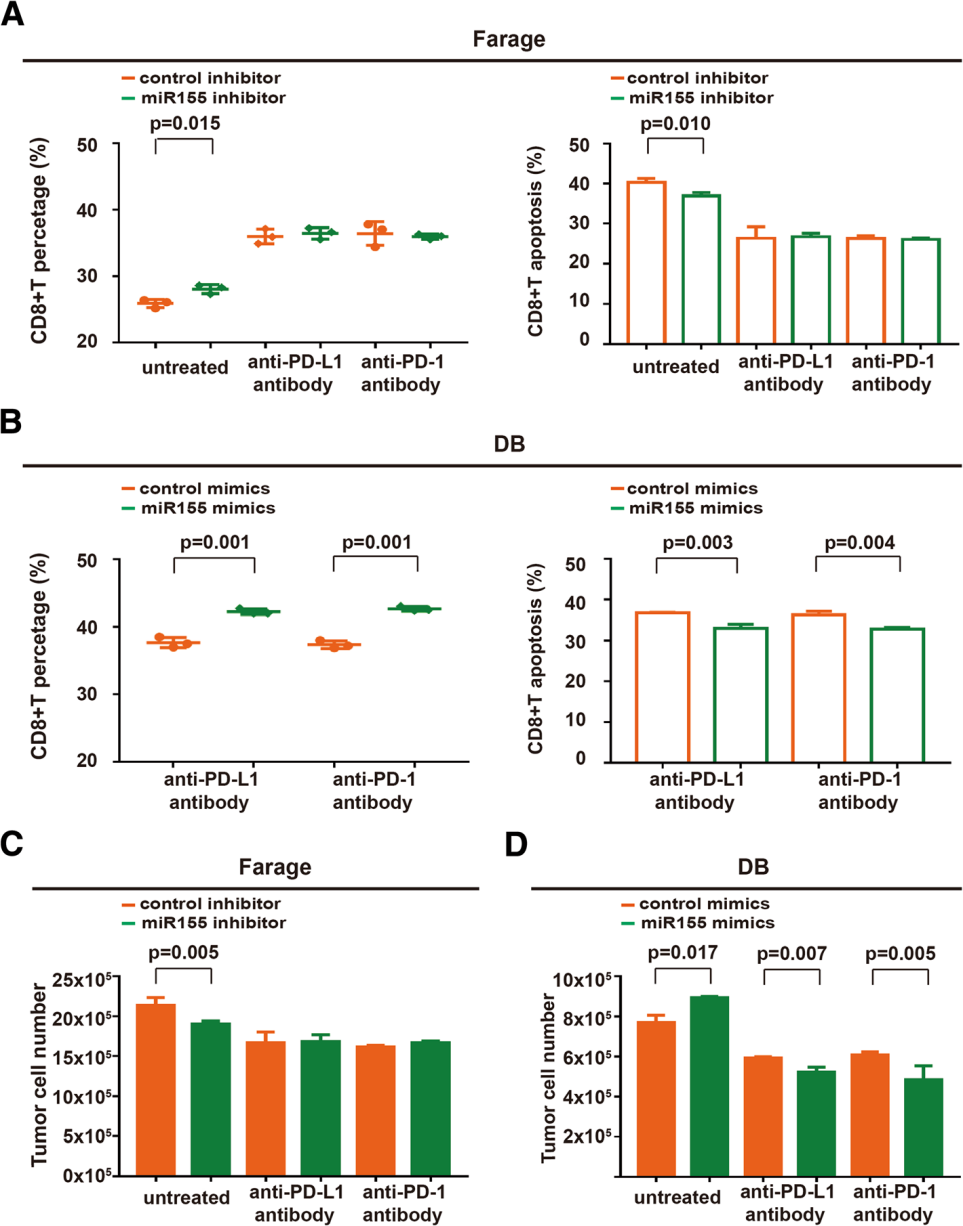
MiR155-mediated CD8+T cell apoptosis was counteracted by PD-1/PD-L1 blockade. **a** CD8+T cells percentage remained constant (left panel) and CD8+T cell apoptosis was not detected (right panel) upon treatment with anti-PD-L1 antibody and anti-PD-1 antibody in the co-culture system of miR155 inhibitor-transfected Farage cells. **b** CD8+T cells percentage was significantly increased (left panel) and CD8+T cell apoptosis was inhibited (right panel) upon treatment with anti-PD-L1 antibody and anti-PD-1 antibody in the co-culture system of miR155 mimics-transfected DB cells. **c** and **d** Farage cell viability remained unchanged in miR155 inhibitor-transfected Farage cells co-culture system treated with anti-PD-L1 and anti-PD-1 antibody (**c**), DB cell viability was obviously reduced by miR155 mimics-transfected DB cells co-culture system treated with anti-PD-L1 and anti-PD-1 antibody (**d**)

The effect of miR155-knockdown Farage cells on CD8+T cell percentage and apoptosis (*P* = 0.015 and *P* = 0.010) was abrogated by anti-PD-L1 antibody and anti-PD-1 antibody in the co-culture systems (Fig. 4A). On the other hand, anti-PD-L1 antibody and anti-PD-1 antibody induced persistence of CD8+T cells and inhibited CD8+T cell apoptosis in the co-culture system of miR155-overexpressing DB cells (*P* = 0.001, P = 0.001, *P* = 0.003, and *P* = 0.004, respectively, Fig. 4B). To further clarify the underlying mechanism of miR155-mediated sensitization of anti-PD-L1 antibody and anti-PD-1 antibody, B-lymphoma cells were sorted from the co-culture systems. When transfected with miR155 inhibitor, Farage cells growth was significantly inhibited (P = 0.005), which were abrogated by anti-PD-L1 antibody and anti-PD-1 antibody (Fig. 4C). Ectopic expression of miR155 in DB cells led to increased sensitivity to anti-PD-L1 antibody and anti-PD-1 antibody (*P* = 0.007 and *P* = 0.005, Fig. 4D). Therefore, miR155 promoted the crosstalk between B-lymphoma cells and CD8+T cells in tumor microenvironment, which could be directly targeted by PD-1/PD-L1 blockade.

### MiR155 modulated PD-1/PD-L1-mediated B-lymphoma cell interaction with CD8+T cells via upregulating PD-L1 expression

**Fig. 5:**
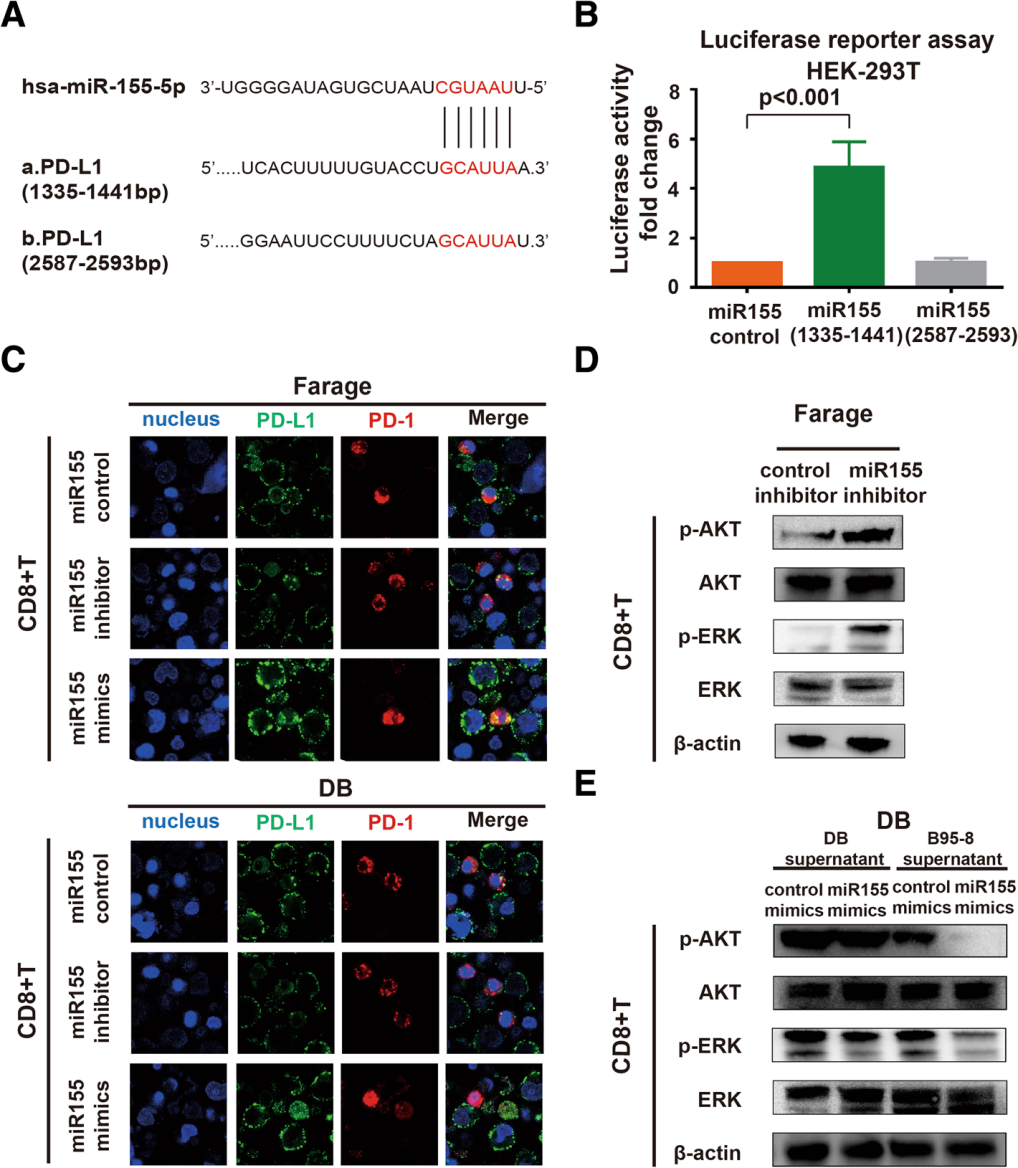
MiR155 modulated PD-1/PD-L1-mediated B-lymphoma cell interaction with CD8+T cells via upregulating PD-L1 expression. **a** Bioinformatics analysis predicted potential binding sites of miR155 with the 3′-UTR of PD-L1. **b** The effect of miR155 on transcriptional activity of PD-L1 3′-UTR was detected by luciferase reporter assay in HEK-293T cells transfected with control mimics or miR155 mimics. **c** PD-1/PD-L1 interaction between lymphoma cells and CD8+T cells in the co-culture systems (Representative immunofluorescene images of PD-L1[green])/PD-1[red] with nucleus counterstained with DAPI [blue]). **d** and **e** Phosphorylated AKT and ERK were detected by western blot in CD8+T cells sorted from the co-culture system of Farage cells (upper panel) and DB cells (lower panel)

Bioinformatics analysis predicted two potential binding sites of PD-L1 3′-UTR with miR155 (Fig. 5A). As revealed by luciferase reporter assay, miR155 positively regulated the transcriptional activity of the PD-L1 3′-UTR (1335-1441 bp) in HEK-293T cells (Fig. 5B), suggesting that miR155 targeted PD-L1 through the 3′-UTR binding site. Immunofluorescence assay showed that PD-1/PD-L1-mediated interaction of B-lymphoma cells with CD8+T cells was inhibited in the co-culture system of miR155-knockdown Farage cells, while enhanced in that of miR155-overexpressing DB cells (Fig. 5C). These results suggested that miR155 could regulate PD-1/PD-L1 interaction between B-lymphoma and CD8+T cells. Previous study reported that PD-1 ligation inhibits activation of T-cell receptor proximal kinases of CD8+T cells via AKT and ERK pathway [13]. Indeed, p-ERK and p-AKT expression of CD8+T cells were upregulated in the co-culture system of miR155-knockdown Farage cells (Fig. 5D) and downregulated in that of miR155-overexpressing DB cells (Fig. 5E).

Exhausted CD8+T cells in chronic EBV infections have sustained activation of PD-1/PD-L1 pathway [14, 15], which could affect downstream AKT and ERK inactivation in CD8+T cells. In according with data from EBV+ DLBCL patients, the effect of miR155 on p-AKT and p-ERK expression of CD8+T cells was reinforced in the co-culture system of EBV-infected DB cells (Fig. 5E). Thus, miR155 could be further enhanced by EBV infection, which induced PD-1/PD-L1 activation in tumor microenvironment through AKT and ERK pathway.

### Anti-PD-L1 antibody exhibited in vivo activity on miR155-overexpressing B-cell lymphoma

**Fig. 6:**
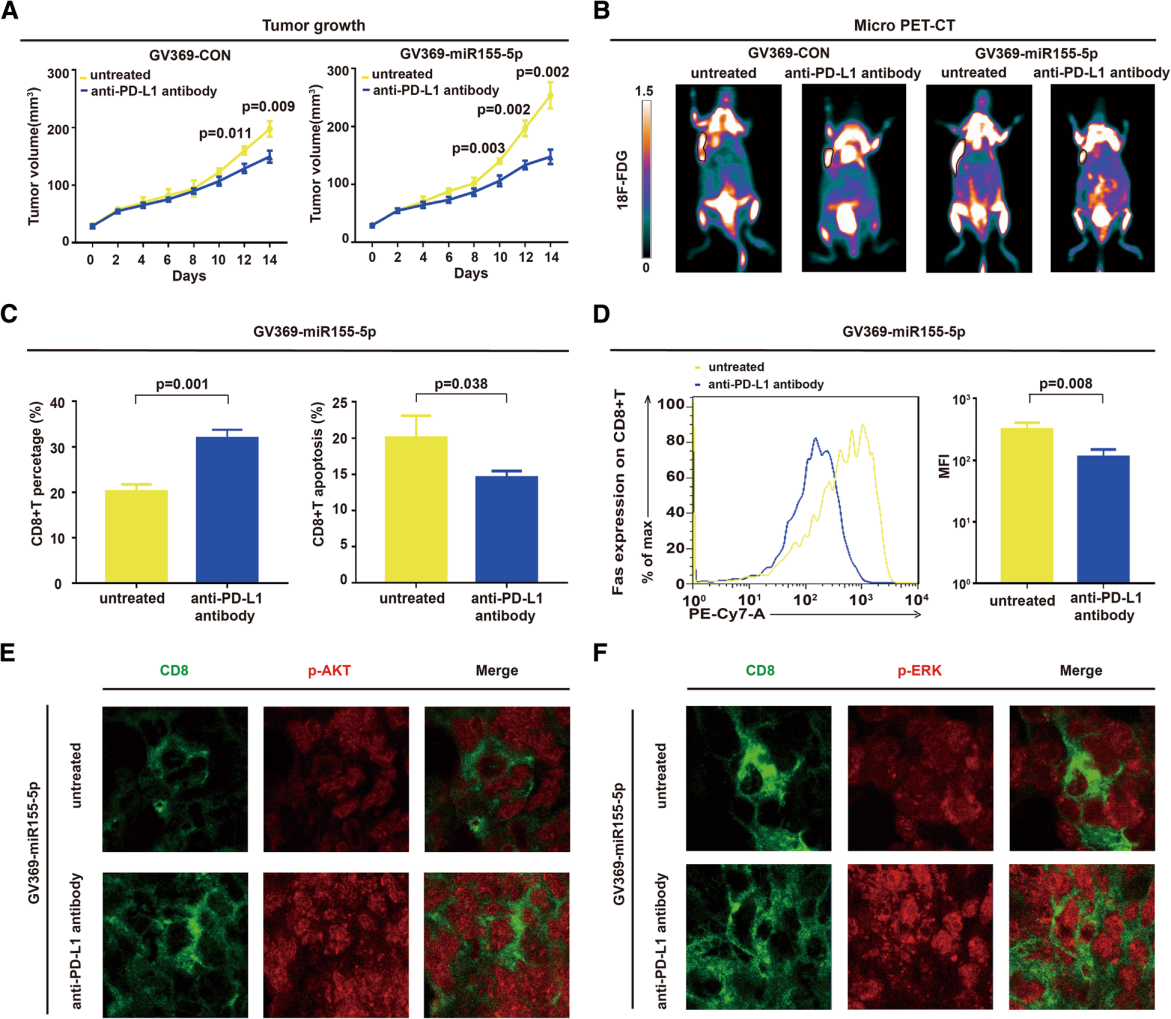
Anti-PD-L1 antibody exhibited in vivo activity on miR155-overexpressing B-cell lymphoma. **a** PD-L1 antibody treatment significantly abrogated miR155-overexpressing GV369-miR155 tumor growth (right panel), comparing to GV369-CON group (left panel). **b** Micro-PET/CT showed increased standardized uptake value intensity in miR155-overexpressing GV369-miR155 tumors and the intensity was significantly decreased by anti-PD-L1 antibody treatment. **c** and **d** CD8+T cell percentage was induced, as well as CD8+T cell apoptosis and Fas expression were inhibited in GV369-miR155 group treated with anti-PD-L1 antibody. **e** and **f** Expression of p-AKT and p-ERK on CD8+T cells was significantly enhanced in GV369-miR155 group treated with anti-PD-L1 antibody

Murine xenograft model was established with subcutaneous injection of A20 cells stably transfected with GV369-CON or GV369-miR155. Comparing with GV369-CON tumors (*P* = 0.011 at Day 12, *P* = 0.009 at Day 14), anti-PD-L1 antibody treatment exhibited an earlier anti-tumor activity on GV369-miR155 tumors (*P* = 0.003 at Day 10, *P* = 0.002 at Day 12, P = 0.002 at Day 14, respectively, Fig. 6A). Further analysis by one-away ANOVA was performed, with *P* < 0.001 presented on Day 12 and Day 14. F-fluorodeoxyglucose small-animal PET/CT was performed to visualize tumors implanted in the flank of nude mice (Fig. 6B). As in vitro study, compared with untreated group, CD8+T cells percentage was increased (*P* = 0.001, Fig. 6C), while CD8+T cell apoptosis (*P* = 0.038, Fig. 6C) and Fas expression were significantly inhibited in GV369-miR155 group upon anti-PD-L1 antibody treatment (*P* = 0.008, Fig. 6D). Both p-AKT and p-ERK expression of CD8+T cells were significantly enhanced with anti-PD-L1 antibody treatment as revealed by immunofluorescence assay (Fig. 6E and F). Anti-PD-L1 antibody also exhibited in vivo activity on GV369-CON B-cell lymphoma (Additional file 1: Figure S1).

## Discussion

MiR155 is critically involved in B-cell lymphoma progression. Experimentally, miR155 transgenic mice exhibit pre-B cell proliferation in spleen and bone marrow, followed by malignant B-cell transformation [16]. Downregulation of miR155 promotes B-lymphoma cell apoptosis and delays xenograft tumor formation in nude mice [17]. In clinical settings, high expression of tumor miR155 predicts treatment failure in 79 patients with DLBCL [12]. Our study confirmed the role of serum miR155 overexpression on poor prognosis in a large cohort of DLBCL. More recently, gene ontology and pathway analysis have suggested that miR155 acts as communicators between tumor cells and microenvironment. In this study, we demonstrated the significant relation of serum miR155 overexpression with inferior survival time, reduced peripheral blood immune cells, and T-cell dysfunction of DLBCL. These findings not only confirmed circulating miR155 as an adverse biomarker derived from lymphoma cells, but also provided a direct link of miR155 to immune suppressive status in DLBCL.

MiR155 contributes to impaired T cell-dependent antibody response [18], promotes production of both monocytic and granulocytic MDSCs and induces chemoresistance through C/EBPβ/IL6/IL6R/STAT3 signaling axis in tumor-associated macrophages [19, 20]. Here both in vitro and in vivo, we provided direct evidence that B-lymphoma cell-derived miR155 specifically modulates Fas-mediated apoptosis of CD8+T cells, indicative an alternative mechanism of miR155 on tumor immune responses. PD-L1 is a co-stimulatory molecule primarily expressed by antigen-presenting cells and subsequently regulates T-cell immune reaction. Binding of PD-L1 to its receptor PD-1 inhibits proliferation of activated T cells, referring as the main therapeutic mechanism of PD-1/PD-L1 blockade [21]. Here in B-lymphoma cells, miR155 upregulated PD-L1 expression through directly binding to the 3′-UTR region, provoked CD8+T cell apoptosis, and inhibited tumor immunity in a PD-1/PD-L1 dependent manner. As mechanism of action, miRNA can bind with AU rich elements located in the 3′-UTR and enhance gene expression [22]. In our study, the 3′-UTR binding site of PD-L1 is typical AU rich elements. In accordance with the fact that miR155 is required by tumor growth and IFN-γ production of T cells within tumor microenvironment [23], our data suggested a functional interaction between tumor cells and CD8+T cells through miR155-PD-L1 regulatory axis, leading to the sensitization of B-lymphoma to anti-PD-L1 antibody. Meanwhile, AKT and ERK phosphorylation are dispensable for CD8+T cell function [24, 25]. MiR155 also altered CD8+T cell function via inducing AKT/ERK dephosphorylation of CD8+T cells, which could be targeted by interrupting PD-1/PD-L1 interaction. Together, although potentially oncogenic, miR155 was a potential target for anti-PD-L1 antibody treatment of B-cell lymphoma.

Importantly, miR155 overexpression was more frequently observed in EBV-positive DLBCL, in according with in vitro study that latent membrane protein-1 of EBV induces the expression of B-cell integration cluster, a precursor form of miR155, in B-lymphoma cells [26]. PD-L1 is upregulated in subsets of virus-associated aggressive B-cell lymphomas including EBV-associated DLBCL, HHV8-associated plasmablastic lymphoma, which may respond to anti-PD-1 antibodies [9, 27, 28]. During monitoring EBV infection, CD8+T cell exhaustion is mediated by tumor cell PD-L1 expression [9]. Restoration of CD8+T cells under chronic virus infection condition was achieved by therapeutic targeting of PD-1/PD-L1 pathway [29]. Key regulation of PD-1-mediated CD8+T cell function were AKT and ERK pathway [13]. Here we showed that miR155-induced alterations of CD8+T cells were particularly obvious in EBV-infected situation, rendering CD8+T cells as a therapeutic mediator of immune checkpoint inhibitors.

## Conclusions

Our findings confirmed the oncogenic potential of miR155 in DLBCL by modulating tumor microenvironment. Although related to tumor progression, miR155 indicated the sensitivity of B-lymphoma cells to anti-PD-L1 antibody. Restoration of CD8+T cell number and function represents a promising clinical strategy in treating EBV-associated lymphoid malignancies.

## Additional file

Additional file 1: **Figure S1.** Anti-PD-L1 antibody exhibited in vivo activity on GV369-CON B-cell lymphoma. A and B: CD8+T cell percentage was enhanced, as well as CD8+T cell apoptosis and Fas expression were inhibited in GV369-CON group treated with anti-PD-L1 antibody. C and D: Expression of p-AKT and p-ERK on CD8+T cells was significantly upregulated in GV369-CON group treated with anti-PD-L1 antibody. (JPG 724 kb)

## Abbreviations

B7-H1: B7 homolog 1
DLBCL: Diffuse large B-cell lymphoma
miRs: MicroRNAs
OS: Overall survival
PD-1: programmed death-1
PD-L1: programmed death ligand 1
PFS: Progression-free survival
