# Supplementary figures and images for: MiR155 sensitized B-lymphoma cells to anti-PD-L1 antibody via PD-1/PD-L1-mediated lymphoma cell interaction with CD8+T cells
Source: Mol Cancer. 2019 Mar 30;18:54. doi: 10.1186/s12943-019-0977-3 (PMC6441197; doi:10.1186/s12943-019-0977-3)

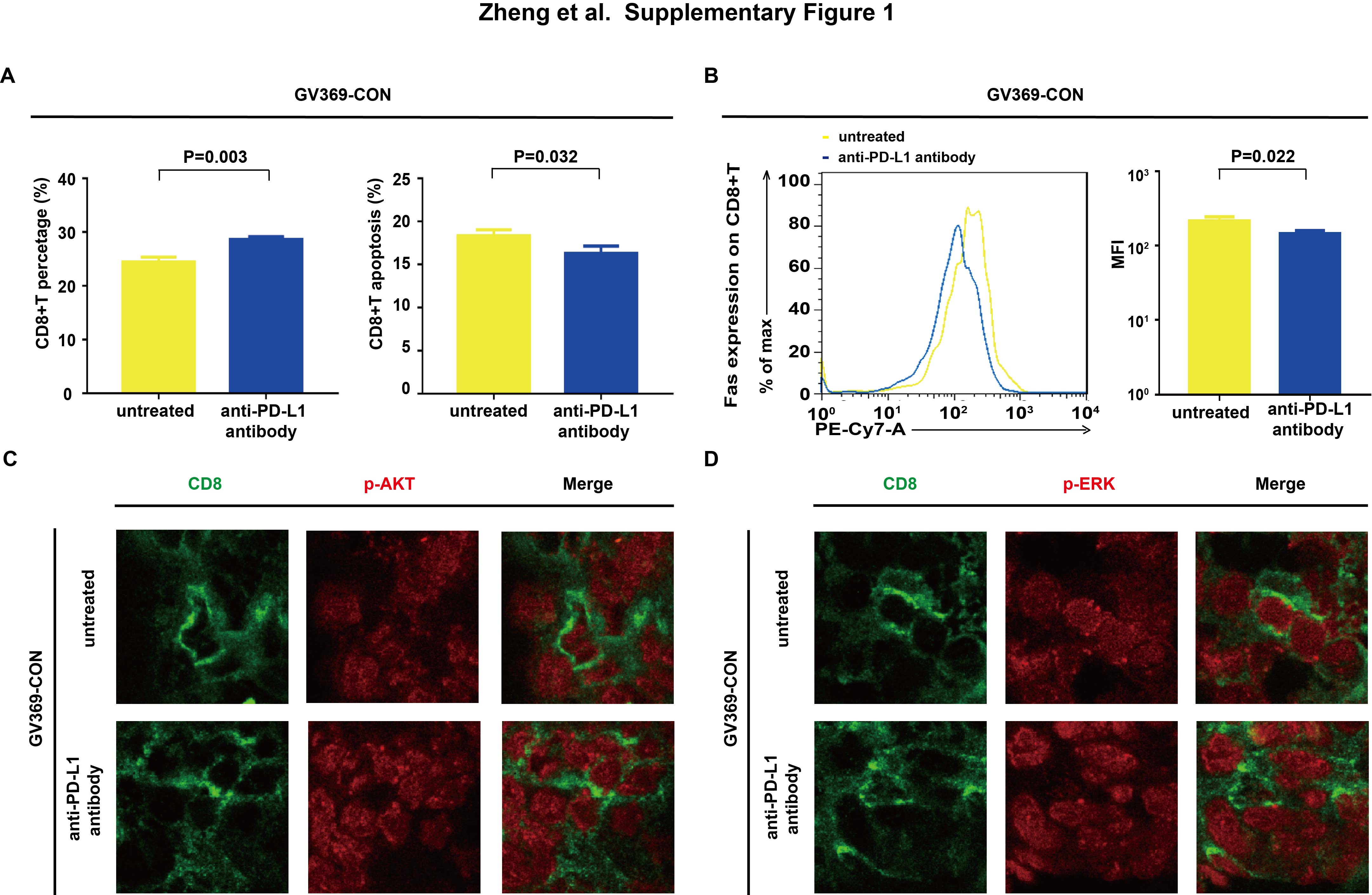

Supplement: Supplementary file 1 — Figure S1. Anti-PD-L1 antibody exhibited in vivo activity on GV369-CON B-cell lymphoma. A and B: CD8+T cell percentage was enhanced, as well as CD8+T cell apoptosis and Fas expression were inhibited in GV369-CON group treated with anti-PD-L1 antibody. C and D: Expression of p-AKT and p-ERK on CD8+T cells was significantly upregulated in GV369-CON group treated with anti-PD-L1 antibody. (JPG 724 kb) [file 12943_2019_977_MOESM1_ESM.jpg]
